# Acetozone in the Treatment of Summer Diarrhea

**Published:** 1904-09

**Authors:** 


### Acetozone in the Treatment of Summer Diarrhea.

In the treatment of any form of diarrhea an accurate diagnosis must first be made. For convenience it is customary to classify diarrheas somewhat after this fashion: (1) diarrhea of relaxation, or serous diarrhea, due to disordered innervation; (2) crapulous or lienteric diarrhea, due to imperfect digestion; (3) catarrhal diarrhea, acute or chronic; and, (4) ulcerative diarrhea, due to intestinal ulceration.

This classification is by no mean perfect, as is shown by the multiplicity of terms applied to the various pathologic states characterised by diarrhea. Thus we have the terms acute inflammatory diarrhea, acute summer diarrhea, choleraic diarrhea, dysenteric diarrhea, nervous diarrhea, tuberculous diarrhea, and the like. In each case the diagnosis is determined by the actual condition prevailing, of which the intestinal laxity is usually but a prominent symptom.

The question of treatment is one of the utmost importance. Without entering into a discussion of what soon proves to be a very broad subject, it may be worth our while to consider briefly the status of the antiseptic method of treating intestinal disorders, especially those caused by pathologic organisms and of which diarrhea is the chief symptom. Apart from well-directed efforts to clear the intestine of bacteria, reduce the temperature, sustain the vitality of the patient, regulate the diet, secure proper hygienic conditions, rest, and good care, the selection of the proper antiseptic agent demands the exercise of the physician's best judgment.

Whether or not it be possible to attain intestinal asepsis is, of course, a debatable question, but it is a well-established clinical fact that intestinal antiseptics do good and modify the course of enteric diseases of bacterial origin, notably typhoid fever, dysentery and summer diarrhea. However, there is a difference in the degree of efficiency of the various antiseptics, the utility of many being limited by the risk of untoward action from excessive dosage. In those cases of ileocolitis caused by the bacillus of Shiga many of the serious symptoms are due to a mixed infection, to combat which prompt and vigorous measures are required.

The experiments of Novy and Freer (Contributions to Medical Research, p. 114) with benzoyl-acetyl-peroxide (acetozone) showed that this substance is extremely germicidal to the organisms found in the alimentary canal. Its administration to rabbits resulted in the "practical sterilisation of the contents of the stomach." In several experiments with these animals "the intestinal tract apart from the cecal pouch, was found to be sterile." Neither bouillon tubes nor agar showed growths, though the controls gave abundant cultures. Other experiments showed that enzymes and toxins are also destroyed or rendered inert by acetozone. Further study demonstrated not only the remarkable germicidal power of acetozone, but also the fact that its aqueous solutions may be given internally, and even injected intravenously, without harm. From these data we infer that this substance ranks among the most powerful germicidal agents, while it exerts no harmful effect upon the human organism, and may, therefore, be employed as a therapeutic agent in the treatment of summer diarrhea and other infectious enteric diseases with the best effect. There seems to be abundant evidence to warrant the suggestion that acetozone solution should prove most valuable in colonic flushing, as it is entirely free from the danger that attends the use of large quantities of even weak solution of mercuric chloride, and for that reason may be used fearlessly.

